# Supplementary material for: A genome-wide association study provides insights into the genetic etiology of 57 essential and non-essential trace elements in humans
Source: Commun Biol. 2024 Apr 9;7:432. doi: 10.1038/s42003-024-06101-z (PMC11004147; doi:10.1038/s42003-024-06101-z)
Supplement: Supplementary file 2 — Description of Additional Supplementary Files [file 42003_2024_6101_MOESM2_ESM.pdf]

Description of Additional Supplementary Files

File name: Supplementary Data 1
Description: Study descriptives.

File name: Supplementary Data 2
Description: Index variants in statistically significant loci (p<5E-8) associated with Trace elements.

File name: Supplementary Data 3
Description: Comparison of meta-analysis index variants across the individual studies.

File name: Supplementary Data 4
Description: Sensitivity analyses in HUNT.

File name: Supplementary Data 5
Description: Protein-altering variants in high LD (correlation R2>0.8 in HUNT) with meta-analysis index variants.

File name: Supplementary Data 6
Description: LD Score Regression SNP heritability.

File name: Supplementary Data 7
Description: Phenome-wide associations (p-value<9.7E-7) between phecodes and trace element meta-analysis index variants in the UK Biobank. Source data for figure 1.

File name: Supplementary Data 8
Description: Phenome-wide associations (p-value<9.7E-7) between trace element meta-analysis index variants and biomarkers in UK Biobank. Source data for figure 1.

File name: Supplementary Data 9
Description: Phenome-wide associations (p-value<9.7E-7) between trace element meta-analysis index variants and continuous variables in UK Biobank. Source data for figure 1.

File name: Supplementary Data 10
Description: Phenome-wide associations (p-value<9.7E-7) between trace element index variants (HUNT only, MAF>0.5%) and biomarkers in UK Biobank.

File name: Supplementary Data 11
Description: Phenome-wide associations (p-value<9.7E-7) between trace element index variants (HUNT only, MAF>0.5%) and continuous traits in UK Biobank.

File name: Supplementary Data 12
Description: Mendelian randomization analysis of the effect of trace elements on health outcomes.
